# Correction: Canadian natural science graduate stipends lie below the poverty line

**DOI:** 10.1371/journal.pone.0331503

**Published:** 2025-09-03

**Authors:** Andrew J. Fraass, Thomas J. Bailey, Kayona Karunakumar, Andrea E. Wishart

The affiliation for the third author is incorrect. The correct affiliation is not indicated. Kayona Karunakumar is not affiliated with #4 but with: Support Our Science, Ottawa, ON, Canada.
