# Supplementary material for: Canadian natural science graduate stipends lie below the poverty line
Source: PLoS One. 2025 May 22;20(5):e0313972. doi: 10.1371/journal.pone.0313972 (PMC12097606; doi:10.1371/journal.pone.0313972)
Supplement: S1 data — (DOCX) [file pone.0313972.s001.docx]

# Supplemental information

## Data collection

In addition to tuition and stipend values as described in the main text, we also gathered the following information for each program: if funding was guaranteed and for how long, if waivers were provided to offset higher international tuition levels, and if teaching assistantship (TA) or research assistantships (RA) roles were required and if so, how many hours.

[Minimum Stipend Data](https://docs.google.com/spreadsheets/d/1STusmXjLqOZeInesHmFcUbIf_eWV9eDwFvfxobQ5LlE/edit?usp=sharing)

Data for this project can be found in multiple formats. A GitHub repository (<https://github.com/UVicMicropaleo/Canadian-Minimum-Graduate-Stipends>) hosts a machine readable file and all code associated with the project. Data collection was performed by sharing a GoogleSheet (<https://docs.google.com/spreadsheets/d/1STusmXjLqOZeInesHmFcUbIf_eWV9eDwFvfxobQ5LlE/edit?usp=sharing>) for all collaborators to use. In order to speed replication of this study across other fields or funding regimes, the authors invite others to mirror the spreadsheet and engage in their own data collection (in collaboration or not).

## Scoring data transparency for tuition and stipends

Tuition Data was scored out of 4 for the level of ease it took to get to the selected program. Here, we worked with links through multiple web pages to obtain course-specific figures, assess how linked it was from the program site or through Google; and locate tuition values on the program site. This scoring system also assessed the level of ease to parse for the following: annual tuition located in one place (i.e., not only accessible by semester); annual fees clearly stated (i.e., if the program/student’s status only needed to be entered one time), and to what extent the information was shown on the website and not found in an external document.

Stipend Data was scored out of 6 for the level of ease it took to get to the web page which included stipend figures. This included assessing the following: if information was or was not available; if it was hard (i.e., if there was an undue amount of clicking required to get to the information); if it was moderate (i.e., consisting of more than two clicks from the program homepage or if the program homepage was slightly more challenging to find); or easy (i.e., consisting of one or two clicks from the program homepage or if the program homepage was slightly harder to locate). This scoring system also assessed to what extent complete information could be found regarding international students, the effect of scholarships, and the net stipend (after tuition).
