# Supplementary material for: Canadian natural science graduate stipends lie below the poverty line
Source: PLoS One. 2025 May 22;20(5):e0313972. doi: 10.1371/journal.pone.0313972 (PMC12097606; doi:10.1371/journal.pone.0313972)
Supplement: S1 Table — Tuition transparency was assessed as ease of parsing, while stipend transparency was assessed as completeness of presented data. Scores were assigned based on the rubrics presented in Supplementary Online Material. Higher values correspond to greater discoverability and transparency, while lower values correspond to lower levels of both. (DOCX) [file pone.0313972.s002.docx]

| **Score item** | **Mean ± SD** |
| --- | --- |
| Tuition (out of 2) |  |
| Discoverability | - 0.7 ± 0.6 |
| Parse | - 1.2 ± 0.6 |
| Stipend (out of 3) |  |
| Discoverability | - 1.6 ± 1.3 |
| Complete | - 0.8 ± 1.1 |

**S1 Table.** Mean scores (± standard deviation) for discoverability and transparency of tuition and stipend data. Tuition transparency was assessed as ease of parsing, while stipend transparency was assessed as completeness of presented data. Scores were assigned based on the rubrics presented in Supplementary Online Material. Higher values correspond to greater discoverability and transparency, while lower values correspond to lower levels of both.
