# Supplementary material for: Canadian natural science graduate stipends lie below the poverty line
Source: PLoS One. 2025 May 22;20(5):e0313972. doi: 10.1371/journal.pone.0313972 (PMC12097606; doi:10.1371/journal.pone.0313972)
Supplement: Supplemental Fig 1 — (DOCX) [file pone.0313972.s003.docx]

**
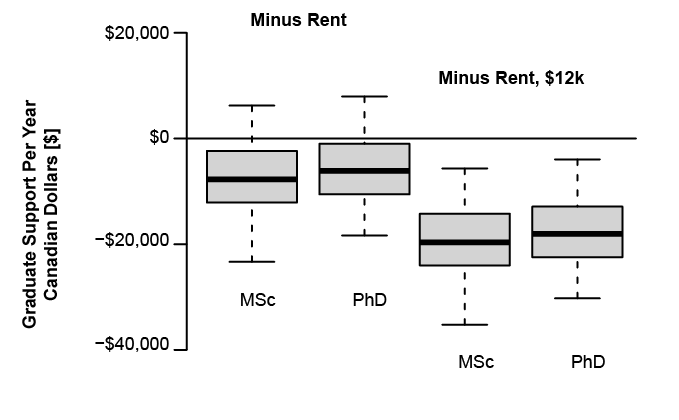
**

**Supplemental Figure 1.** Supported domestic minimum stipends compared to Rentals.ca values. Boxes at left are Net Minimum Stipends minus a local assessment of rents from Rentals.ca [[13](https://paperpile.com/c/xY498C/7DQ4)], while boxes at left subtract an additional $12k to account for food and other costs.
