# Supplementary material for: Canadian natural science graduate stipends lie below the poverty line
Source: PLoS One. 2025 May 22;20(5):e0313972. doi: 10.1371/journal.pone.0313972 (PMC12097606; doi:10.1371/journal.pone.0313972)
Supplement: Supplemental Fig 2 — (DOCX) [file pone.0313972.s004.docx]

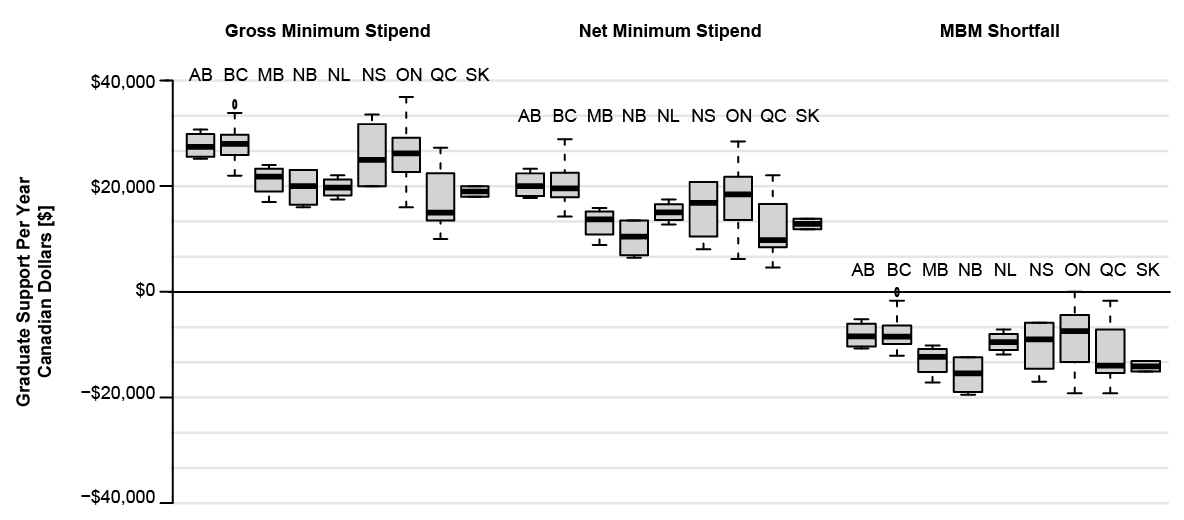


**Supplemental Figure 2.** Supported domestic minimum stipends divided by province. Gross Minimum Stipend (GMS) is the guaranteed funding for a student provided by the institution. Net Minimum Stipend (NMS) is the GMS minus tuition and fees for an institution. MBM Shortfall is the NMS minus the Market Basket Measure (MBM) for an institution's location.
