# Supplementary material for: Canadian natural science graduate stipends lie below the poverty line
Source: PLoS One. 2025 May 22;20(5):e0313972. doi: 10.1371/journal.pone.0313972 (PMC12097606; doi:10.1371/journal.pone.0313972)
Supplement: Supplemental Fig 3 — (DOCX) [file pone.0313972.s005.docx]

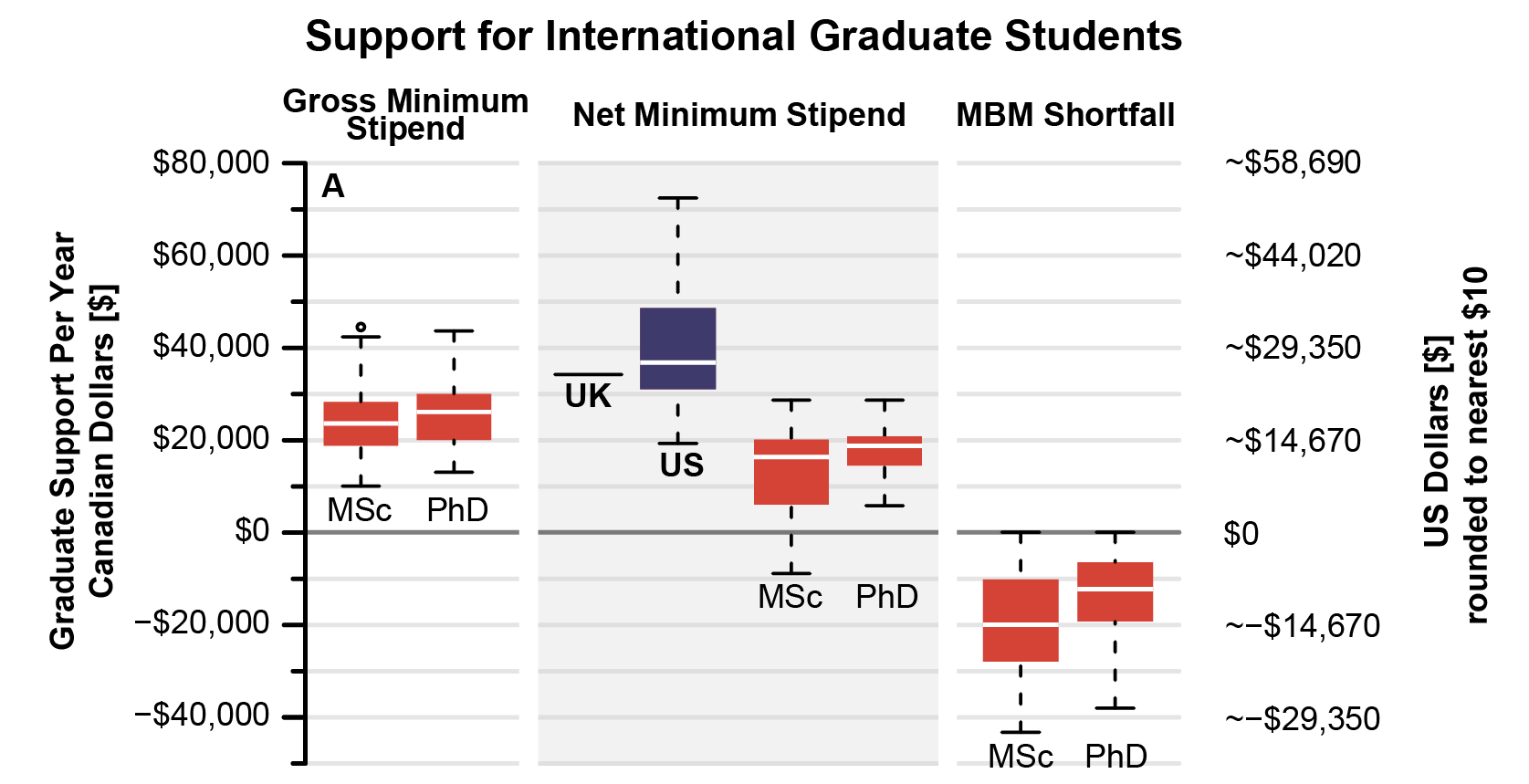


**Supplemental Figure 3.** Annual financial support in Canadian and US dollars to international graduate students (MSc and PhD in physics and biology). Gross Minimum Stipend (GMS) is the guaranteed minimum funding for an MSc or PhD student provided by the institution. Net Minimum Stipend (NMS) is the GMS minus tuition and fees for an institution. MBM Shortfall is the NMS minus the Market Basket Measure (MBM), a poverty threshold, for an institution's location. US Biology stipends are from [[12]](https://paperpile.com/c/xY498C/X4Sw) and should be considered a rough approximation of the US stipends, without accounting for fees or taxes (see text).
