# Supplementary material for: Canadian natural science graduate stipends lie below the poverty line
Source: PLoS One. 2025 May 22;20(5):e0313972. doi: 10.1371/journal.pone.0313972 (PMC12097606; doi:10.1371/journal.pone.0313972)
Supplement: S2 Table — 1.1.26[17]. SEM = Standard error of the mean; SD = Standard deviation. Asterisk * denotes significance at α = 0.01. (DOCX) [file pone.0313972.s006.docx]

|  | **Estimate ± SEM** | **d.f.** | ***t*** | ***p*** |
| --- | --- | --- | --- | --- |
| **Fixed effects** |  |  |  |  |
| Intercept | -8578.0 ± 4069.26 | 22.12 | -2.11 | 0.047 * |
| Endowment  (log_10_ transformed) | 8587.4 ± 1452.8 | 19.88 | 5.91 | < 0.001* |
| Program (reference group: MSc) | 1829.3 ± 525.5 | 54.78 | 3.48 | < 0.001* |
| Field (reference group: Biology) | 870.2 ± 579.1 | 60.20 | 1.50 | 0.138 |
| **Random effects** | **Variance ± SD** |  |  |  |
| University:Province (Intercept) | 9488650 ± 3080 |  |  |  |
| Province (Intercept) | 3984869 ± 1996 |  |  |  |
| Residual | 5661384 ± 2379 |  |  |  |

**S2 Table.** Summary of generalized linear mixed model fit for Net Minimum Stipend (NMS) as a function of university endowment using package lme4 v. 1.1.26 [17]. SEM = Standard error of the mean; SD = Standard deviation. Asterisk * denotes significance at α = 0.01.
