# Supplementary material for: Canadian natural science graduate stipends lie below the poverty line
Source: PLoS One. 2025 May 22;20(5):e0313972. doi: 10.1371/journal.pone.0313972 (PMC12097606; doi:10.1371/journal.pone.0313972)
Supplement: S3 Table — 1.1.26 [17]. SEM = Standard error of the mean; SD = Standard deviation. Asterisk * denotes significance at α = 0.01. (DOCX) [file pone.0313972.s007.docx]

|  | **Estimate ± SEM** | **d.f.** | ***t*** | ***p*** |
| --- | --- | --- | --- | --- |
| **Fixed effects** |  |  |  |  |
| Intercept | -21564.0 ± 7358.6 | 22.44 | -2.93 | 0.008* |
| University Expenses  (log_10_ transformed) | 12273.8 ± 2468.2 | 21.93 | 4.97 | < 0.001* |
| Program (reference group: MSc) | 1829.4 ± 525.5 | 54.78 | 3.48 | < 0.001* |
| Field (reference group: Biology) | 833.2 ± 581.8 | 59.30 | 1.43 | 0.157 |
| **Random effects** | **Variance ± SD** |  |  |  |
| University:Province (Intercept) | 11642290 ± 3412 |  |  |  |
| Province (Intercept) | 6565074 ± 2562 |  |  |  |
| Residual | 5660699 ± 2379 |  |  |  |

**S3 Table.** Summary of generalized linear mixed model fit for Net Minimum Stipend (NMS) as a function of university expenses using package lme4 v. 1.1.26 [17]. SEM = Standard error of the mean; SD = Standard deviation. Asterisk * denotes significance at α = 0.01.
