# Supplementary material for: Canadian natural science graduate stipends lie below the poverty line
Source: PLoS One. 2025 May 22;20(5):e0313972. doi: 10.1371/journal.pone.0313972 (PMC12097606; doi:10.1371/journal.pone.0313972)
Supplement: Attachment — Submitted filename: PONE D-24-49 review.docx [file pone.0313972.s008.docx]

PONE D-24-49-49904

It is difficult to discern the difference between advocacy and research in this paper. I do not know what PLOS ONE’s editorial position is regarding the difference. If advocacy is within the journal’s mandate, with revision the paper perhaps warrants publication. As research it does not.

The most constructive way to explain this is to say that, like other papers of this kind it would toward the end of section on “limitations.” There are several.

1 Sponsored research is funded so differently in the U.S. and U.K. that the validity of evidence from those jurisdictions and comparisons to them are questionable. Perhaps less so for Australia. This makes me think about the findings of studies about policy diffusion and policy dissonance. There is a lot of unacknowledged dissonance here.

2 I appreciate the difficulty of getting data from only two departments, only minimum stipend values, and only NSERC, but as a matter of fact the simplification that the study admits fails to support most of the study’s conclusions. It makes the research a case study. Case studies, by definition, have little capability for generalization.

3 At the beginning the paper makes this assertion: *Graduate students are a primary engine of scientific work for the academic system.* And at the end we find this assertion as if it is a finding of the study: *Graduate students, both in Canada and around the world, are the engine on which academic science runs.*These are remarkable claims that, in my view, are more advocacy than research. As I read the Larivière paper it does not support the primacy that the paper gives to graduate student research in the first instance and the study itself provides no evidence in the second instance.

4	The paper’s first conclusion is about the “hidden curriculum” of an admissions process that results in under-representation in the academy. That may or may not be true, but it is an entirely different research question from where the paper begins, and it is hard to see how the study’s research methodology (which the paper presents very well) leads to that conclusion.

5	The discussion of proportionality is something like the “hidden curriculum” conclusion. The evidentiary basis of the conclusion is not clear, but more to the point it begs a question of an alternative that the paper does not admit: that the problem may be as much or more the number of stipends than the size of the stipends.

6 The study is correct to recognize the differences in stipend tax status between the U.S. and Canada (and maybe the UK, for which the study evidently did not collect comparable information. FYI Like Canada, stipends are not taxed in the UK.) This means that for comparative purposes the net minimum stipend in Canada is higher than the minimum stipend *per se.* This does not mean that Canadian stipends are inadequate and possibly still below the poverty line. It means that the comparative disparity may not be as great as presumed.

7 At several points the study refers to “substantial student debt” carried by graduate students. There are two potentially serious oversights here. First, approximately half of undergraduates in Canada graduate without debt. This varies from province to province. Second, the second oversight is the Canadian Education Tax Credit, which is available to graduates with or without debt.

8 Figure 2 is not conclusive. To be so would require a separation of restricted and unrestricted funds. Restricted means that a donor made a gift for a purpose specified – hence *restricted* – by the donor. Universities must comply with restrictions as long as they are not illegal. Of course, universities can refuse to accept restrictions. So, it is only the unrestricted portion of an endowment that can be directed to stipends. The study perhaps should come to different conclusion: that research-intensive universities in their fund-raising should place a higher priority on stipends.
